# Role of Interleukin‐10 Overexpressing Mesenchymal Stem Cells in Promoting Bone Marrow Functional Recovery in Aplastic Anemia Mice

**DOI:** 10.1155/sci/2300675

**Published:** 2026-08-12

**Authors:** Qinglin Mo, Lixuan Chen, Yingnan Wu, Zhaolin Chen, Fangfang Lin, Yang Xiao, Zenghui Liu

**Affiliations:** ^1^ Translational Medicine Center, The Second Affiliated Hospital of Guangzhou Medical University, Guangzhou, Guangdong, China, gzhmc.edu.cn; ^2^ Immune Cell Laboratory, Shenzhen Qianhai Shekou Pilot Free Trade Zone Hospital, Shenzhen, Guangdong, China; ^3^ Department of Hematology, Shenzhen Qianhai Shekou Pilot Free Trade Zone Hospital, Shenzhen, China; ^4^ The First Affiliated Hospital of Guangzhou University of Chinese Medicine, Guangzhou 510405, China, gzhmc.edu.cn

**Keywords:** aplastic anemia, IL-10, immunoregulation, mesenchymal stem cells

## Abstract

**Background:**

Mesenchymal stem cells (MSCs) are multipotent, nonhematopoietic progenitors capable of supporting hematopoiesis and regulating immunity. Clinical studies have shown that MSCs can aid hematopoietic recovery and balance T helper 17 (Th17) and Treg cells in some aplastic anemia (AA) patients refractory to immunosuppressive therapy (IST). However, full clinical efficacy remains unachieved. To enhance MSC immune regulation while ensuring safety, previous research found interleukin‐10 (IL‐10), an immunosuppressive factor, can boost MSC function. In this study, we used lentiviral transduction to induce IL‐10 gene overexpression in human umbilical cord‐derived MSCs and validated their efficacy and safety in AA mouse models.

**Methods:**

MSCs were isolated from umbilical cords and genetically modified via lentiviral transduction to overexpress IL‐10, generating OEIL10‐MSCs. Empty vector plasmids (pCDH‐CMV‐MCS‐EF1‐copGFP) were transduced into MSC to create PCDH‐MSCs. Both in vitro and in vivo experiments were conducted to investigate their functional changes and mechanisms. Mouse models of nonsevere AA (NSAA) and severe AA (SAA) were established and treated with OEIL10‐MSCs or control PCDH‐MSCs. The therapeutic efficacy of OEIL10‐MSCs in AA was evaluated by assessing blood cell counts, Th17/Treg ratios, and bone marrow histopathology in an AA mouse model. Safety was assessed through liver and kidney function tests.

**Results:**

In vitro experiments verified that OEIL10‐MSCs exhibited enhanced proliferation capacity, improved anti‐inflammatory ability, and reduced oxidative stress levels. In NSAA mouse models, OEIL10‐MSCs were more effective in restoring hematopoietic function and elevating Treg cell proportions. In contrast, in SAA mouse models, OEIL10‐MSCs demonstrated superior efficacy in elevating red blood cell (RBC) levels and reducing DNA damage compared to PCDH‐MSCs.

**Conclusion:**

Overexpression of IL‐10 may enhance the efficacy of MSCs in treating AA, presenting a promising therapeutic strategy for the clinical application of MSCs.

## 1. Introduction

Acquired aplastic anemia (AA) is a rare and potentially lethal disease with evident immune system dysfunction, characterized by pancytopenia and hypoplastic bone marrow [1, 2]. Clinically, acquired AA can be classified into nonsevere AA (NSAA) or severe AA (SAA), with the latter displaying lower blood and bone marrow counts and a higher mortality rate [3]. Moreover, nearly 15% of patients with SAA will eventually develop malignant diseases [4]. Allogeneic hematopoietic stem cell transplantation (allo‐HSCT) is nearly the only curative treatment option for SAA patients. Unfortunately, the widespread application of allo‐HSCT for AA patients is restricted by several factors, such as matching and transplant complications [5, 6]. Immunosuppressive therapy (IST) is the main treatment for AA patients who are ineligible for allo‐HSCT. Nonetheless, approximately 30% of patients do not respond to it, and almost 30%–45% of AA patients will relapse after IST [7–9]. Accordingly, existing therapies have exhibited significant shortcomings, and the major obstacle in AA treatment continues to be refractory or relapsed diseases. A new treatment strategy that is more effective, safer, and more universally applicable is urgently required.

Mesenchymal stem cells (MSCs), the major cellular components of the bone marrow microenvironment, are nonhematopoietic stem cells with functions such as self‐renewal, multipodency, hematopoietic support, and immunoregulation [10]. MSC infusion is a promising method for AA treatment and has been identified as a promising alternative source for allo‐HSCT. Our previous studies found that combining MSCs with allo‐HSCT for SAA patients could promote hematopoietic engraftment and reduce the severity of graft versus host disease (GvHD) [11, 12] However, only 33% of relapsed/refractory AA patients responded to MSCs combined with cyclosporin A (CsA) [13]. The therapeutic efficiency of primary MSCs is limited and insufficient to achieve the desired results. Therefore, specific gene modification of MSCs is being developed to enhance the therapeutic efficiency.

Interleukin‐10 (IL‐10) is a cytokine that was initially found to be secreted by Th2 cells [14]. Subsequent studies have shown that IL‐10 can be secreted by a variety of cells and has anti‐inflammatory and immunosuppressive functions [15]. Qu et al. [16] found that IL‐10 secreted by MSCs could activate signal transducer and activator of transcription 5 (STAT5) and upregulate suppressor of cytokine signaling 3 (SOCS3), resulting in the blockade of T helper 17 (Th17) cell differentiation in vitro. Huber et al. [17] found that specifically blocking the IL‐10 signaling pathway of T cells could induce an increase in Th17 cells. Moreover, studies have shown that the level of IL‐10 in the serum of AA patients is lower than that in the healthy population, and the IL‐10 expression increases significantly when AA patients recover [18–20]. Gao et al. [18] found that most of the differentially expressed genes were associated with immune responses, inflammatory responses, and cell migration between IL‐10‐modified MSCs and unmodified MSCs, which is consistent with the biological functions of IL‐10. In addition, animal experiments showed that transplantation of IL‐10‐modified MSCs could enhance immunosuppressive and anti‐inflammatory functions in spinal cord injury mice. However, the role of IL‐10‐modified MSCs in the treatment of AA remains unknown.

In this study, we examined the functional differences between primary MSCs and OEIL10‐MSCs through in vitro experiments. We further established immune‐mediated mouse models of AA to investigate the role and potential treatment mechanism of OEIL10‐MSCs.

## 2. Results

### 2.1. Identification of UC‐MSCs, OEIL10‐MSCs, and PCDH‐MSCs

**Figure 1:**
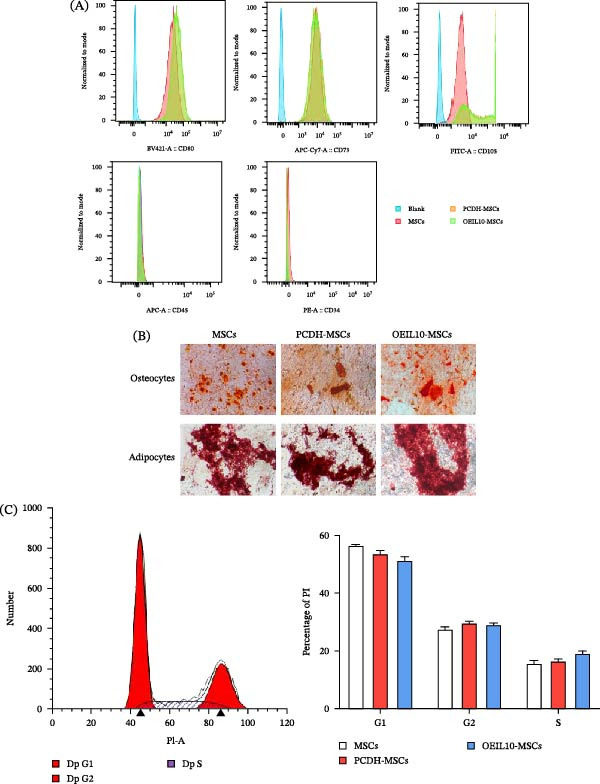
Identification of UC‐MSCs (A) Flow cytometric analysis revealed that UC‐MSCs, PCDH‐MSCs, and OEIL10‐MSCs expressed the positive markers mesenchymal lineage (CD73, CD90, and CD105) and were negative for hematopoietic and endothelial markers (CD34 and CD45). (B) UC‐MSCs, PCDH‐MSCs, and OEIL10‐MSCs demonstrated differentiation potential into adipocytes and osteocytes, as evidenced by staining with Oil Red O and Alizarin Red, respectively. Scale bar = 200 μm. (C) Cell cycle analysis of UC‐MSCs (*n* = 3), PCDH‐MSCs (*n* = 3), and OEIL10‐MSCs (*n* = 3) was conducted using flow cytometry. Two‐tailed Student’s *t*‐test was used for comparisons between two groups.

Cloned spheres were formed from umbilical cords after approximately 3–7 days. The cells exhibited a fibroblast‐like morphology, and the surface markers of the 5th‐generation cells were identified using flow cytometry. The results showed that the mesenchymal markers of CD73, CD90, and CD105 were positive, while the hematopoietic cell surface markers of CD34 and CD45 were negative (Figure 1A). Moreover, the results of alizarin red staining and oil red O staining confirmed that these cells could be successfully induced to differentiate into adipocytes and osteoblasts (Figure 1B). The above characteristics indicate that the cells we isolated are typical UC‐MSCs.

In addition, the immunophenotype and differentiation potential of OEIL10‐MSCs and PCDH‐MSCs remained unchanged after gene modification (Figure 1A,B). Furthermore, there were no significant differences in their cell cycles (Figure 1C).

### 2.2. Proliferation, Anti‐Inflammatory, and Reactive Oxygen Species (ROS)‐Reducing Abilities Were Enhanced in OEIL10‐MSCs

**Figure 2:**
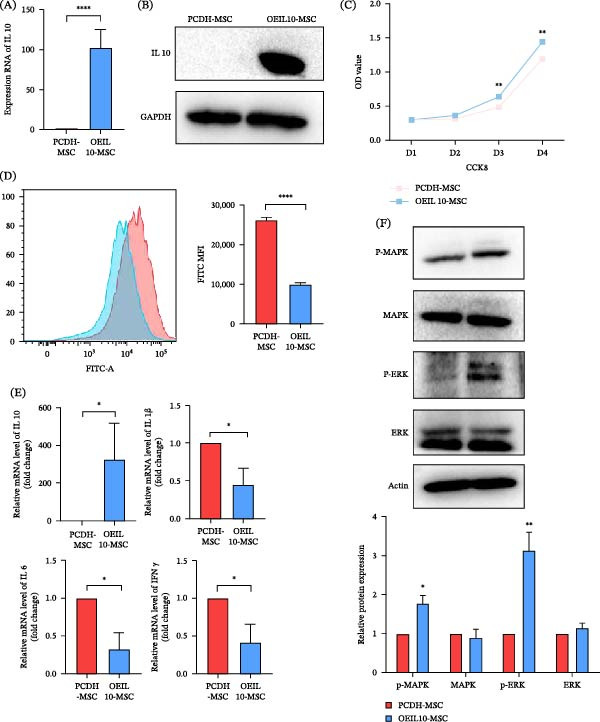
Phenotypic and functional alterations of OEIL10‐MSCs. (A) OEIL10‐MSCs exhibited overexpression of IL‐10 mRNA (each group, *n* = 4). (B) OEIL10‐MSCs demonstrated overexpression of IL‐10 protein. (C) Representative proliferative curves for PCDH‐MSCs and OEIL10‐MSCs are displayed (each group, *n* = 3). (D) ROS levels were detected using the ROS probe DCFH‐DA on a flow cytometer. Representative graphs and statistical analyses are presented (each group, *n* = 3). (E) Levels of inflammatory factors in peripheral blood mononuclear cells (PBMCs) cultured with supernatants from PCDH‐MSCs and OEIL10‐MSCs were assessed (each group, *n* = 4). (F) The MAPK/ERK pathway was activated in OEIL10‐MSCs (each group, *n* = 3). Significance levels indicated as follows:  ^∗^ *p*  < 0.05,  ^∗∗^ *p*  < 0.01,  ^∗∗∗^ *p*  < 0.001,  ^∗∗∗∗^ *p* < 0.0004. Two‐tailed Student’s *t*‐test was used for comparisons between two groups.

To assess the lentiviral transduction efficiency of OEIL10‐MSCs, we compared the phenotypes and functions between OEIL10‐MSCs and PCDH‐MSCs. As shown in Figure 2A,B, OEIL10‐MSCs significantly overexpressed the IL‐10 gene at both the RNA and protein levels. In vitro, the proliferation ability of OEIL10‐MSCs was enhanced (Figure 2C). In addition, compared with PCDH‐MSCs, the ROS level of OEIL10‐MSCs was significantly downregulated (Figure 2D). The supernatant from OEIL10‐MSCs cultures was cocultured with peripheral blood mononuclear cells (PBMCs) to evaluate their anti‐inflammatory properties. The findings revealed that OEIL10‐MSCs were capable of enhancing the RNA expression of IL‐10 while simultaneously suppressing the RNA expression of IL‐6, IL‐1β, and IFN‐γ, as illustrated in Figure 2E. Notably, IL‐6 plays a crucial role in promoting the differentiation of Th17 cells, whereas IL‐1β, in synergy with inflammatory cytokines such as IL‐17, amplifies tissue damage. Conversely, IL‐10 is a key cytokine secreted by Treg cells, whereas IFN‐γ inhibits the differentiation and function of Treg cells. These alterations in cytokine levels suggest that OEIL‐10 MSCs have the potential to inhibit Th17 cells, stimulate Treg cell production, and thereby rectify the imbalance between Th17 and Treg cells in autoimmune diseases such as AA.

Previous studies [18, 19] have shown that the mitogen‐activated protein kinase (MAPK)/extracellular signal‐regulated kinases (ERK) pathway is involved in physiological and pathological processes such as cell proliferation, growth, inflammation, and ROS. Consequently, in our experiments, we also investigated the MAPK/ERK pathway and observed its activation in OEIL10‐MSCs, as shown in Figure 2F. Our findings suggest that activation of the MAPK/ERK pathway may mediate the enhanced functions observed in OEIL10‐MSCs.

### 2.3. OEIL10‐MSCs Enhance Immunosuppressive Function to Restore Dysregulated Hematopoiesis in NSAA Mice

**Figure 3:**
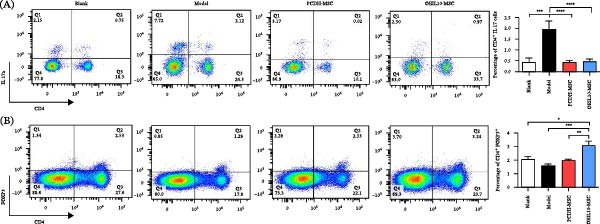
Treg and Th17 cell subsets in NSAA mice from all groups. (A) The proportions of CD4^+^IL‐17a^+^ Th17 cells among the different groups of NSAA mice are presented (blank group, *n* = 5; model group, *n* = 7; PCDH‐MSC, *n* = 10; OEIL10‐MSC, *n* = 10). (B) The proportions of CD4^+^FOXP3^+^ Treg cells among the various groups of NSAA mice are shown. Representative graphs and statistical analyses are provided (blank group, *n* = 3; model group, *n* = 4; PCDH‐MSC, *n* = 4; OEIL10‐MSC, *n* = 4). Significance levels are indicated as follows:  ^∗∗^ *p*  < 0.01,  ^∗∗∗^ *p*  < 0.001,  ^∗∗∗∗^ *p* < 0.0004. One‐way ANOVA was used for comparisons among multiple groups, with Bonferroni correction as a post hoc test.

To establish mouse models of AA, low‐dose (3.0 Gy) or high‐dose (5.0 Gy) X‐ray irradiation was administered, and the mice were subsequently classified as either NSAA or SAA based on disease progression assessed through blood cell counts and bone marrow histopathology [21, 22]. Both NSAA and SAA mice exhibited pancytopenia and hypoplastic bone marrow, which served as indicators for evaluating the treatment efficacy. Notably, SAA mice had white blood cell (WBC) counts below 0.5 × 10^9^/L and a 50% reduction in hematopoietic cells. Furthermore, T cells isolated from the spleens of these mice revealed a significant downregulation of Treg cell proportions in NSAA mice, accompanied by a clear upregulation of Th17 cell proportions (Figure 3A,B).

**Figure 4:**
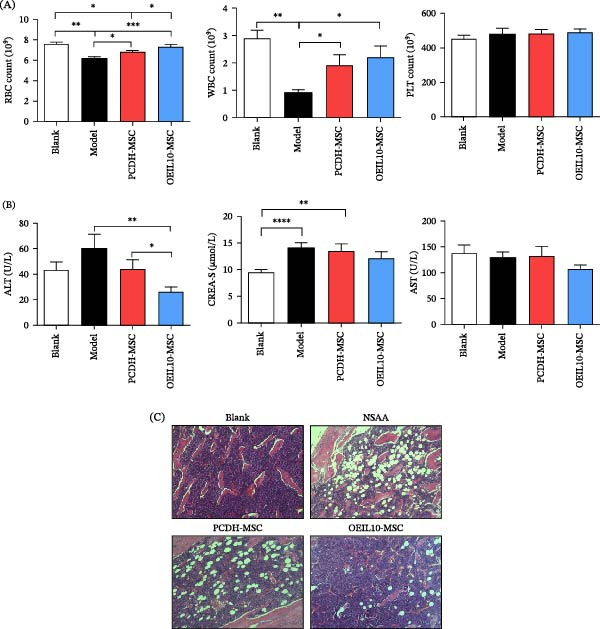
Analysis of peripheral blood and bone marrow in NSAA mice from all groups. (A) Blood cell counts for mice from all groups are presented. (B) Levels of aspartate aminotransferase (AST), alanine aminotransferase (ALT), and creatinine (CREA) in mice from all groups are shown (blank group, *n* = 4; model group, *n* = 6; PCDH‐MSC, *n* = 6; OEIL10‐MSC, *n* = 6). (C) Representative hematoxylin and eosin (H&E) staining of bone marrow sections from each group is displayed (blank group, *n* = 4; model group, *n* = 6; PCDH‐MSC, *n* = 6; OEIL10‐MSC, *n* = 6). Significance levels are indicated as follows:  ^∗^ *p*  < 0.05,  ^∗∗^ *p*  < 0.01,  ^∗∗∗^ *p*  < 0.001,  ^∗∗∗∗^ *p* < 0.0004. One‐way ANOVA was used for comparisons among multiple groups, with Bonferroni correction as a post hoc test.

After treatment for 2 weeks, mice with NSAA that received either PCDH‐MSCs or OEIL10‐MSCs exhibited varying degrees of recovery from the disease. Specifically, the PCDH‐MSCs and OEIL10‐MSCs groups demonstrated significantly improved recovery in WBC and red blood cell (RBC) counts in the peripheral blood, as well as a substantial improvement in the recovery of dysplastic bone marrow in comparison with the model group (Figure 4A,C). The OEIL10‐MSCs group demonstrated a marked increase in the number of peripheral blood RBC and a significant decrease in alanine aminotransferase (ALT) levels when compared to the PCDH‐MSCs group (Figure 4A,B). Additionally, there was a substantial increase in the proportion of Treg cells in the spleens (Figure 3B). Based on these observations, we hypothesize that OEIL10‐MSCs may enhance hematopoiesis in the bone marrow of NSAA mice by potentiating the immune regulatory function.

### 2.4. OEIL10‐MSCs Ameliorate Bone Marrow Failure and Mitigate DNA Damage, Yet Fail to Enhance Blood Cell Counts in SAA Mice

**Figure 5:**
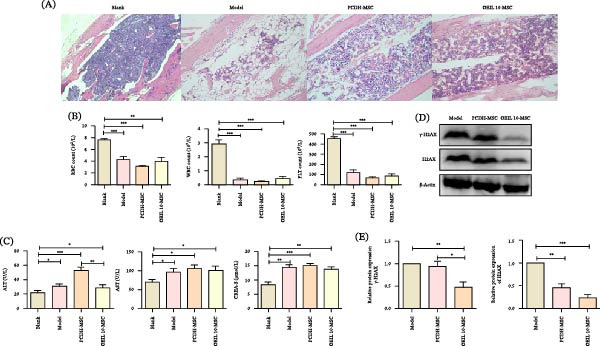
Therapeutic effect in SAA mice from all groups. (A) Representative hematoxylin and eosin (H&E) staining of bone marrow sections from SAA mice in all groups is displayed. (B) Blood cell counts for SAA mice from all groups are presented. (C) Levels of aspartate aminotransferase (AST), alanine aminotransferase (ALT), and creatinine (CREA) in SAA mice from all groups are shown (blank group, *n* = 4; model group, *n* = 6; PCDH‐MSC, *n* = 6; OEIL10‐MSC, *n* = 6). (D) Proteins of H2AX and γ‐H2AX in the spleens of SAA mice from different groups were detected by Western blotting (each group, *n* = 3). (E) Relative protein expression levels of H2AX are presented (each group, *n* = 3). Significance levels are indicated as follows:  ^∗^ *p* < 0.05,  ^∗∗^ *p* < 0.01,  ^∗∗∗^ *p* < 0.001. Two‐tailed Student’s *t*‐test was used for comparisons between two groups. One‐way ANOVA was used for comparisons among multiple groups, with Bonferroni correction as a post hoc test.

SAA mice exhibited severe disease manifestations, including trilineage dysplasia, significantly impaired liver and kidney functions, and bone marrow failure, as evidenced by routine blood analysis, biochemical indices, and bone marrow histopathological examination. Despite notable recovery from bone marrow failure in the treated group (Figure 5A), infusion of MSCs failed to restore blood cell counts or liver and kidney functions (Figure 5B,C). There was no statistically significant difference between the OEIL10‐MSCs group and the PCDH‐MSCs group in terms of blood cell counts (Figure 5B).

Interestingly, although no significant improvement in blood cell count was observed in the SAA group, we found that the OEIL10‐MSCs group exhibited a significant reduction in the levels of H2A histone family member X (H2AX) and γ‐H2AX proteins (markers of DNA damage) compared to the PCDH‐MSCs group and the model group (Figure 5D,E). This finding suggests that OEIL10‐MSCs may have the potential to mitigate DNA damage.

### 2.5. Safety Evaluation of OEIL10‐MSCs in Major Organs of Mice

**Figure 6:**
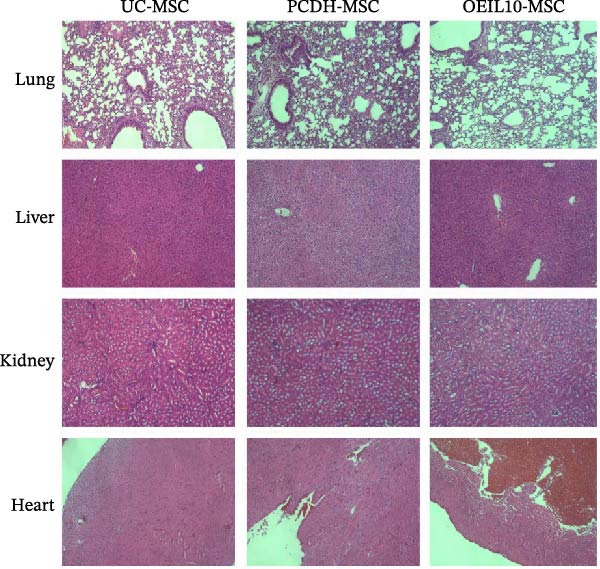
Histological and safety assessment of OEIL10‐MSC in major organs.Histological evaluation of major organs (heart, lung, liver, and kidney) collected from mice two weeks after intravenous infusion of OEIL10‐MSC. Tissue sections were stained with hematoxylin and eosin (H&E) for histopathological assessment. H&E staining revealed no significant tissue damage or inflammatory infiltration in any of the examined organs. Importantly, no tumorigenicity was observed in any of the organs, indicating a favorable safety profile of OEIL10‐MSC administration.

After 2 weeks of OEIL10‐MSC infusion, the major organs of the mice, including the heart, lung, liver, and kidney, were collected to assess their safety. The histological examinations of these organs are presented in the Figure 6. Notably, the major organs of mice treated with OEIL10‐MSCs did not exhibit any tumorigenic changes, confirming the safety of OEIL10‐MSCs.

## 3. Discussion

The immune dysregulation of the hematopoietic microenvironment plays a vital role in the pathogenesis of AA. As the main stromal component of bone marrow, bone marrow‐derived MSCs dysfunction performs both trigger and helper functions in AA. Thus, MSCs infusion is considered a promising advance for AA treatment. However, the therapeutic effects of primary MSCs are limited due to inconsistent cell characteristics, irregular regimens, deviated doses, and different infusion modes [12, 23].

In this study, the IL‐10 gene of UC‐MSCs was overexpressed to improve the therapeutic effect. In vitro, we verified that OEIL10‐MSCs showed a lower oxidative stress level than that of PCDH‐MSCs, as well as increased proliferation and anti‐inflammatory abilities. In addition, the MAPK/ERK signaling pathway was activated in UC‐MSCs by overexpressing the IL‐10 gene, and the activation of the MAPK/ERK pathway is speculated to play a crucial role in improving the functions of immunosuppression and anti‐inflammatory of OEIL10‐MSCs [19]. The current research results on whether OEIL10‐MSCs can promote proliferation are controversial. Our study and others [12, 24] have shown that OEIL10‐MSCs can promote proliferation, while Zuo et al.’s [19] study suggests that they cannot.

A previous study revealed MSCs infused therapy might have the potential tumorigenic risk [25]. In this study, we also assessed the safety of OEIL10‐MSCs after the infusion of MSCs into AA mouse models. In agreement with the results [25], our results showed that OEIL10‐MSCs did not increase the oncogenic risk in vivo. Subsequently, we treated AA mice with OEIL10‐MSCs to evaluate their efficacy in vivo. Our findings showed that bone marrow hematopoiesis was notably restored by PCDH‐MSCs and OEIL10‐MSCs in NSAA and SAA mice. Of these, OEIL10‐MSCs had better efficacy in NSAA mice. Consistent with previous studies [26, 27], the development of NSAA mice was accompanied by an increase of Th17 cells and a decrease of Treg cells. However, the proportion of Th17 cells decreased and Tregs increased after infusion of PCDH‐MSCs and OEIL10‐MSCs. Among these, the upregulation of the Treg cell proportion was more pronounced. IL‐10 is an immunomodulatory and anti‐inflammatory cytokine, which is a key factor and contributes to immune homeostasis in mediating immunosuppression of MSCs. Multiple studies [28–30] have shown that in autoimmune disease, IL‐10‐modified MSCs could better downregulate the proliferation and differentiation of Th1 and Th17 cells while upregulating the proportion of Treg cells to Th2 cells, which are immunosuppressive cells. Therefore, we consider that IL‐10‐modified UC‐MSCs could improve treatment efficiency in NSAA mice by boosting immunosuppressive function.

In addition, we found that protein expressions of γ‐H2AX and H2AX were markedly increased but significantly declined after OEIL10‐MSCs treatment in SAA mice. As we know, AA patients have a high risk of developing myelodysplastic syndromes (MDS) under stress conditions [31], with the latter accompanied by a high frequency of mutations in spliceosome genes [32, 33]. Zhu et al. [34] reported that genes with altered isoform usage in AA patients were significantly enriched in DNA repair, and AA and MDS patients exhibited similar defects in alternative splicing that were primarily associated with DNA repair, suggesting an unstable genomic state in AA patients. DNA damage and repair may represent a critical mechanism of AA progression to MDS. Thus, it may be speculated that OEIL10‐MSCs rescued SAA mice by decreasing DNA damage and may have the potential to prevent malignant progression.

There are several methods to improve the therapeutic effects of primary MSCs. For instance, exosomes extracted from MSCs supernatant and gene‐modified MSCs are used to enhance therapeutic efficacy [35–37]. Moreover, haploidentical HSCs combined with allogeneic MSCs have been successfully applied in clinical transplantation for AA patients [38, 39].

We acknowledge that the mechanistic insights of the present study remain limited. Although we observed activation of the MAPK/ERK pathway in IL‐10‐overexpressing UC‐MSCs, the downstream signaling mechanisms and specific molecular interactions underlying the enhanced immunoregulatory and anti‐inflammatory effects were not fully explored. For instance, how MAPK/ERK activation regulates the expression of immunosuppressive factors, modulates oxidative stress, or interacts with other potential pathways (e.g., NF‐κB or PI3K/Akt) requires further investigation. Future studies employing targeted inhibitors, gene silencing, or proteomic analyses are needed to delineate the complete signaling network. This limitation does not detract from our main findings but warrants further investigation to better understand the therapeutic mechanisms of IL‐10‐modified MSCs in AA.

Our results offer a new perspective for treating AA, showing that MSCs overexpressing IL‐10 could enhance their functions and improve therapeutic efficacy. Furthermore, this finding provides substantial data support for the clinical utilization of MSCs.

## 4. Conclusion

In conclusion, our results showed that OEIL10‐MSCs had promising therapeutic efficacy in AA, although the detailed mechanism remains to be further studied.

## 5. Materials and Methods

### 5.1. Isolation, Culture, and Identification of UC‐MSCs

The umbilical cord of the newborn was collected from fully informed healthy human volunteer donors with the written consent of their guardians. In brief, the umbilical cord was cut into 3 mm sections and digested with 2 µg/mL collagenase A at 37°C for 30 min and then washed thoroughly using precooled PBS. Tissue sections were cultured in 10 cm cell culture dishes using a specific culture medium (TBD, China) for UC‐MSCs, supplemented with 1% 100 U/mL streptomycin sulfate and 100 U/mL penicillin G in a humidified atmosphere with 5% CO_2_ at 37°C. After 3 days, the medium was first changed, and the cells were passaged when the cell density reached 90%. UC‐MSCs in Passage 5 were used for differentiation and surface marker detection.

For identification, cells were collected and incubated with mouse anti‐human CD34, CD45, CD73, CD90, and CD105 flow antibodies (Biolegend) on ice for 30 min. Subsequently, MSC‐positive (CD73, CD90, and CD105) and MSC‐negative (CD34 and CD45) surface marker expression was analyzed by flow cytometry in primary MSCs and OEIL10‐MSCs. The differentiation ability of adipocytes and osteoblasts was detected using an OriCell osteogenic stimulatory kit (Caygen, China) and a MesenCult adipogenic differentiation kit (Stemcell, Canada).

### 5.2. UC‐MSCs Were Transduced With Lentivirus to Overexpress the IL‐10 Gene

Lentiviruses were produced by co‐transfecting HEK293T cells with the IL‐10 overexpression plasmid or empty pCDH‐CMV‐MCS‐EF1‐copGFP vector (purchased from Tsingke Biotechnology Co., Ltd.) together with packaging plasmids (psPAX2 and pMD2.G) using a standard calcium phosphate transfection method. Viral supernatants were collected at 48 and 72 h posttransfection, pooled, filtered (0.45 µm), and concentrated by ultracentrifugation. The final viral titer was determined to be 1 × 10^7^ TU/mL. At the fourth passage, UC‐MSCs were used for lentiviral transduction with IL‐10 lentivirus or PCDH‐vector lentivirus (Tsingke Biotechnology Co., Ltd). In brief, UC‐MSCs were seeded in 6‐well plates, IL‐10 lentivirus, PCDH‐ vector lentivirus, and 6 µg/mL polybrene were added, respectively (multiplicity of infection [MOI] = 10 and viral titer = 1 × 10^7^ TU/mL). The medium was changed after 8–12 h. After 48–72 h, lentiviral transduction efficiency was validated by quantitative real‐time polymerase chain reaction (qRT‐PCR) and western blot.

### 5.3. Cell Counting Kit‐8 (CCK‐8) Assay

Cell viability was evaluated by the CCK‐8 assay (Dojindo, Japan). OEIL10‐MSCs or PCDH‐MSCs were seeded in 96‐well plates (5 × 10^3^ cells/well). At the indicated time points (days 1, 2, 3, and 4), CCK‐8 solution diluted with medium (1:10) was added and incubated at 37°C for 1 h. Next, absorbance was detected at 450 nm using a microplate reader (Bio‐Rad, model 550).

### 5.4. Cell Cycle Detection and ROS Measurement

OEIL10‐MSCs or PCDH‐MSCs were seeded in 6‐well plates and cultured for 48 h. The 2’,7’‐dichlorodihydrofluorescein diacetate (DCFH‐DA, Beyotime, Reactive Oxygen Species Assay Kit, S0033S, China) was serially diluted in PBS to a final concentration of 10 µM. The cells were then collected and resuspended in the diluted DCFH‐DA solution at a concentration of 1 × 10^6^ cells/mL. The cells were then incubated in a cell incubator at 37°C for 15 min. The mixture was inverted every 3–5 min to ensure sufficient contact between the probe and the cells. The cells were washed three times with PBS to ensure the complete removal of noninternalized DCFH‐DA. Then, the samples were resuspended in 1 mL of PBS. The FITC fluorescent channel was used to detect ROS levels by flow cytometry. The resulting data were analyzed with FlowJo software.

For cell cycle detection, 1 × 10^6^ cells were collected, and the cell culture medium was removed by centrifugation. The cells were washed once with PBS, and the supernatant was removed by centrifugation. Then, 1 mL of DNA staining solution and 10 µL of Permeabilization solution (MULTI SCIENCES, Cell Cycle Staining Kit, CCS012, China) were added, followed by thorough mixing by vortexing and shaking for 5–10 s. The cells were then incubated at room temperature in the dark for 30 min. After three washes with PBS, the cells were resuspended in 1 mL of PBS and examined using a flow cytometer. The resulting data were analyzed with FlowJo software.

### 5.5. PBMCs Were Cocultured With UC‐MSCs‐Conditioned Medium to Examine the Anti‐Inflammation Ability

Peripheral blood was obtained from healthy donors. It was diluted twice in sterile PBS and mixed with an equal volume of human lymphocyte separation medium to obtain PBMCs by density gradient centrifugation. The UC‐MSCs‐conditioned medium was collected from OEIL10‐MSCs or PCDH‐MSCs and centrifuged at 1500 rpm for 5 min first. Subsequently, PBMCs were seeded in 12‐well plates and cultured in RPMI‐1640 medium and the conditioned medium from OEIL10‐MSCs or PCDH‐MSCs (1:1 volume ratio), supplemented with 10% FBS and 10 µg/mL PHA. Relative RNA expressions of inflammation‐related genes were detected by qRT‐PCR.

### 5.6. Quantitative Real‐Time PCR

Total RNA was isolated from PBMCs using the BIOG Microdose Cell RNA Kit (Zhao Rui Biotech, China) and reverse transcribed into cDNA using the HiScript III RT SuperMix for qPCR kit (Vazyme, China). The primers for qRT‐PCR were listed in Table 1. The housekeeping gene GAPDH was used to normalize gene expression. Each group was tested in at least triplicate.

**Table 1:** Primer sequence for qRT‐PCR.

Gene	Primer sequence
H‐ACTIN F	CATGTACGTTGCTATCCAGGC
H‐ACTIN R	CTCCTTAATGTCACGCACGAT
H‐GAPDH F	GGAGCGAGATCCCTCCAAAAT
H‐GAPDH R	GGCTGTTGTCATACTTCTCATGG
H‐IL‐10 F	GACTTTAAGGGTTACCTGGGTTG
H‐IL‐10 R	TCACATGCGCCTTGATGTCTG
H‐IL1B F	AGCTACGAATCTCCGACCAC
H‐IL1B R	CGTTATCCCATGTGTCGAAGAA
H‐IL4 F	CGGCAACTTTGTCCACGGA
H‐IL4 R	TCTGTTACGGTCAACTCGGTG
H‐IL6 F	ACTCACCTCTTCAGAACGAATTG
H‐IL6 R	CCATCTTTGGAAGGTTCAGGTTG
H‐IFNG F	TCGGTAACTGACTTGAATGTCCA
H‐IFNG R	TCGCTTCCCTGTTTTAGCTGC
H‐TNFA F	GAGGCCAAGCCCTGGTATG
H‐TNFA R	CGGGCCGATTGATCTCAGC

### 5.7. Western Blotting

Western blotting was performed as described previously [22] with minor modifications. Briefly, equal protein amounts were separated by SDS‐PAGE and transferred onto PVDF membranes. After blocking, membranes were incubated overnight at 4°C with primary antibodies against γ‑H2AX (ET1602‑2, HUABIO), H2AX (ET1705‑97, HUABIO), IL‑10 (ab34843, Abcam), MAPK (66234‑1‑Ig, Proteintech), ERK (11257‑1‑AP, Proteintech), p‑ERK (80031‑1‑RR, Proteintech), and p‑MAPK (28796‑1‑AP, Proteintech). After washing, membranes were incubated with HRP‑conjugated secondary antibodies. Finally, the expression of each protein was measured by a ChemiSignal^TM^ ECL Plus kit (Qinxiang Biotechnology, China) and the density of protein bands was quantified using ImageJ software.

### 5.8. Establishment and Treatment of Immune‐Mediated AA Mouse Models

Male C57BL/6N mice and male DBA/2 mice at 8–10 weeks of age were purchased from Charles River (Beijing, China) and housed in cages under pathogen‐free conditions. Animals were kept at an average temperature of 22–24°C, following 12 h light–dark cycles. All mice utilized in our experiments were approved by the Guangzhou Institute of Biomedicine and Health, Chinese Academy of Sciences (Approval Number N2021060).

DBA/2 mice were used as donors for the lymph node collection. Single‐cell lymphocyte suspensions were prepared and resuspended in PBS. First, C57 6N mice were exposed to 3.0 Gy or 5.0 Gy X‐ray irradiation (MultiRad), and they were infused with 1 × 10^6^ lymphocytes (resuspended in 200 µL PBS) via tail vein within 4 h to establish immune‐mediated AA mouse models. At the same time, the same volume of PBS was injected into C57 6N mice in the blank control group. On day 3, AA mice were treated with primary UC‐MSCs, PCDH‐MSCs, or OEIL10‐MSCs, respectively. Mice in each treated group were injected with 2 × 10^6^ cells resuspended in 200 µL PBS. The control group was treated with the same dose of PBS. Fourteen days later, after anesthesia and the collection of peripheral blood, all mice were sacrificed for efficacy evaluation by blood cell counts, AST, ALT, creatinine, and bone marrow histopathology.

### 5.9. Flow Cytometry

Lymphocytes were isolated from the spleens of mice using the Mouse Spleen Lymphocyte Separation Solution Kit (TBD). The lymphocytes of each mouse were divided into two parts. One part of the lymphocytes was stimulated with PMA (Beyotime, 1000×) and BFA (Biolegend, 1000×) for 6h, and then incubated with anti‐mouse CD4 antibody (Biolegend) on ice for 30 min. Subsequently, they were incubated with anti‐mouse IL‐17 antibody (Biolegend) on ice for 30 min after they were fixed and permeabilized by the Cyto‐Fast Fix/Perm Buffer Set (Biolegend). The rest part of the lymphocytes was incubated directly with anti‐mouse CD4 antibody on ice for 30 min and then incubated with anti‐mouse FOXP3 antibody on ice for 30 min after fixation by the FOXP3 Fixation/Permeabilization Buffer kit (Biolegend). Eventually, the proportion of Th17 and Treg cells of mouse spleens was detected by flow cytometry.

### 5.10. Histopathology

The experiment was performed according to a previous study [40]. The femurs and tissues of mice were fixed in 10% formalin overnight, femurs were decalcified with EDTA decalcification solution firstly, and then femurs and tissues were dehydrated in an alcohol gradient, embedded in paraffin, sliced, and stained by H&E staining for evaluation of therapeutic effect and tumorigenicity. Images were taken with an Olympus IX71 (Japan).

### 5.11. Statistical Methods

All data were analyzed with SPSS 25.0 and GraphPad Prism 5.0. A two‐tailed Student’s *t*‐test was used for comparisons between two groups. One‐way ANOVA was used for comparisons among multiple groups, with Bonferroni correction as a post hoc test. A *p*‐value <0.05 was considered statistically significant.

**Nomenclature**
UC‐MSCs: Human umbilical cord derived‐mesenchymal stem cells
AAA: Plastic anemia
NSAA: Nonsevere aplastic anemia
SAA: Severe aplastic anemia
OEIL10‐MSCs: MSCs which was overexpressed IL‐10
allo‐HSCT: Allogeneic hematopoietic stem cell transplantation
MSCs: Mesenchymal stem cells
GvHD: Graft versus host disease
CsA: Cyclosporin A
IL‐10: Interleukin 10
PBMCs: Peripheral blood mononuclear cells
H2AXH2A: Histone family member X
SOCS3: Suppressor of cytokine signaling 3
STAT5: Signal transducer and activator of transcription 5
ROS: Reactive oxygen species
IST: Immunosuppressive therapy
MAPK: Mitogen‐activated protein kinase
ERK: Extracellular regulated protein kinases
WBC: White blood cell
RBC: Red blood cell
MDS: Myelodysplastic syndromes.

## Author Contributions

Qinglin Mo conducted the culture and identification of mesenchymal stromal cells, performed RT‐PCR, WB, and FCM, conducted statistical analysis, designed the study, and drafted the manuscript. Additionally, Qinglin Mo, Lixuan Chen, and Yingnan Wu were involved in establishing animal models and carrying out the treatments. Lixuan Chen, Zhaolin Chen, and Fangfang Lin contributed to the study design and assisted in drafting and editing the manuscript. Yingnan Wu also participated in statistical analysis and FCM. Yang Xiao and Zenghui Liu were responsible for the overall study design. Zenghui Liu was responsible for revising and polishing the manuscript.

## Funding

This work was supported by funding from the National Natural Science Foundation of China (Project Numbers 82474238 and 81873426), the “Guben” Project for Enhancing the Capability of First‐Level Disciplines at the Guangzhou University of Chinese Medicine (Project Number GZY2025GB0108), the Shenzhen Basic Research Special Project (Natural Science Foundation) Basic Research General Program (Project Number JCYJ20220530151613030), and the Shenzhen Nanshan District Technology Development and Creative Design Project (Project Number NS2022004).

## Disclosure

All authors have reviewed and approved the final version of the manuscript.

## Ethics Statement

Informed consent was obtained from the donors, and ethics approval was obtained according to the guidelines of the Guangzhou Institute of Biomedicine and Health, Chinese Academy of Sciences. All the procedures described in this report have been approved by the Guangzhou Institute of Biomedicine and Health, Chinese Academy of Science (Approval Number N2021060; approval date: April 2021).

## Consent

The authors have nothing to report.

## Conflicts of Interest

The authors declare no conflicts of interest.

## Data Availability

The datasets used and/or analyzed during the current study are available from the corresponding author upon reasonable request.

## References

[1] Young N. S. , Aplastic Anemia, The New England Journal of Medicine. (2018) 379, no. 17, 1643–1656. 30354958 10.1056/NEJMra1413485 PMC6467577

[2] DeZern A. E. and Churpek J. E. , Approach to the Diagnosis of Aplastic Anemia, Blood Advances. (2021) 5, no. 12, 2660–2671, 10.1182/bloodadvances.2021004345. 34156438 PMC8270669

[3] Fattizzo B. , Giannotta J. A. , and Barcellini W. , Mesenchymal Stem Cells in Aplastic Anemia and Myelodysplastic Syndromes: The “Seed and Soil” Crosstalk, International Journal of Molecular Sciences. (2020) 21, no. 15, 10.3390/ijms21155438, 5438. 32751628 PMC7432231

[4] Sun L. and Babushok D. V. , Secondary Myelodysplastic Syndrome and Leukemia in Acquired Aplastic Anemia and Paroxysmal Nocturnal Hemoglobinuria, Blood. (2020) 136, no. 1, 36–49, 10.1182/blood.2019000940. 32430502 PMC7332901

[5] Gurnari C. , Pagliuca S. , Prata P. , and etal H. , Clinical and Molecular Determinants of Clonal Evolution in Aplastic Anemia and Paroxysmal Nocturnal Hemoglobinuria, Journal of Clinical Oncology. (2023) 41, no. 1, 132–142, 10.1200/JCO.22.00710. 36054881 PMC10476808

[6] Zhang Y. , Liang Y. , and Zhang X. , et al.Pre-Transplant Platelet Refractoriness and Alternative Donors are Associated With Cytomegalovirus Retinitis in Hematopoietic Stem Cell Transplantation for Severe Aplastic Anemia, Frontiers in Cellular and Infection Microbiology. (2022) 12, 10.3389/fcimb.2022.870296, 870296. 35372094 PMC8964998

[7] Scheinberg P. , Acquired Severe Aplastic Anaemia: How Medical Therapy Evolved in the 20th and 21st Centuries, British Journal of Haematology. (2021) 194, no. 6, 954–969, 10.1111/bjh.17403. 33855695

[8] Bacigalupo A. , How I Treat Acquired Aplastic Anemia, Blood. (2017) 129, no. 11, 1428–1436, 10.1182/blood-2016-08-693481. 28096088

[9] Georges G. E. , Doney K. , and Storb R. , Severe Aplastic Anemia: Allogeneic Bone Marrow Transplantation as First-Line Treatment, Blood Advances. (2018) 2, no. 15, 2020–2028, 10.1182/bloodadvances.2018021162. 30108110 PMC6093726

[10] Mishra V. K. , Shih H.-H. , and Parveen F. , et al.Identifying the Therapeutic Significance of Mesenchymal Stem Cells, Cells. (2020) 9, no. 5, 10.3390/cells9051145, 1145. 32384763 PMC7291143

[11] Liu Z. , Zhang Y. , and Xiao H. , et al.Cotransplantation of Bone Marrow-Derived Mesenchymal Stem Cells in Haploidentical Hematopoietic Stem Cell Transplantation in Patients With Severe Aplastic Anemia: An Interim Summary for a Multicenter Phase II Trial Results, Bone Marrow Transplantation. (2017) 52, no. 5, 704–710, 10.1038/bmt.2016.347. 28067873

[12] Liu Z. , Wu X. , and Wang S. , et al.Co-Transplantation of Mesenchymal Stem Cells Makes Haploidentical HSCT a Potential Comparable Therapy With Matched Sibling Donor HSCT for Patients With Severe Aplastic Anemia, Therapeutic Advances in Hematology. (2020) 11, 10.1177/2040620720965411, 154257187. PMC7605036 33194162

[13] Xiao Y. , Jiang Z.-J. , and Pang Y. , et al.Efficacy and Safety of Mesenchymal Stromal Cell Treatment From Related Donors for Patients With Refractory Aplastic Anemia, Cytotherapy. (2013) 15, no. 7, 760–766, 10.1016/j.jcyt.2013.03.007. 23731760

[14] Fiorentino D. F. , Bond M. W. , and Mosmann T. R. , Two Types of Mouse T Helper Cell. IV. Th2 Clones Secrete a Factor That Inhibits Cytokine Production by Th1 Clones, The Journal of Experimental Medicine. (1989) 170, no. 6, 2081–2095, 10.1084/jem.170.6.2081. 2531194 PMC2189521

[15] Mollazadeh H. , Cicero A. F. G. , Blesso C. N. , Pirro M. , Majeed M. , and Sahebkar A. , Immune Modulation by Curcumin: The Role of Interleukin-10, Critical Reviews in Food Science and Nutrition. (2017) 59, no. 1, 89–101, 10.1080/10408398.2017.1358139. 28799796

[16] Qu X. , Liu X. , Cheng K. , Yang R. , and Zhao R. C. H. , Mesenchymal Stem Cells Inhibit Th17 Cell Differentiation by IL-10 Secretion, Experimental Hematology. (2012) 40, no. 9, 761–770, 10.1016/j.exphem.2012.05.006. 22634392

[17] Huber S. , Gagliani N. , and Esplugues E. , et al.Th17 Cells Express Interleukin-10 Receptor and are Controlled by Foxp3? and Foxp3^+^ Regulatory CD4^+^ T Cells in an Interleukin-10-Dependent Manner, Immunity. (2011) 34, no. 4, 554–565, 10.1016/j.immuni.2011.01.020. 21511184 PMC3113617

[18] Gao T. , Huang F. , and Wang W. , et al.Interleukin-10 Genetically Modified Clinical-Grade Mesenchymal Stromal Cells Markedly Reinforced Functional Recovery After Spinal Cord Injury via Directing Alternative Activation of Macrophages, Cellular & Molecular Biology Letters. (2022) 27, no. 1, 10.1186/s11658-022-00325-9, 27. 35300585 PMC8931978

[19] Zuo Y. , Lu X. , and Wang X. , et al.High-Dose Aluminum Exposure Further Alerts Immune Phenotype in Aplastic Anemia Patients, Biological Trace Element Research. (2021) 199, no. 5, 1743–1753, 10.1007/s12011-020-02313-6. 32761514 PMC7990755

[20] Yan S. , Zhang C. , and Ji X. , et al.MSC-ACE2 Ameliorates *Streptococcus uberis*-Induced Inflammatory Injury in Mammary Epithelial Cells by Upregulating the IL-10/STAT3/SOCS3 Pathway, Frontiers in Immunology. (2022) 13, 10.3389/fimmu.2022.870780, 870780. 35677060 PMC9167935

[21] Seyfried A. N. , McCabe A. , Smith J. N. P. , Calvi L. M. , and MacNamara K. C. , CCR5 Maintains Macrophages in the Bone Marrow and Drives Hematopoietic Failure in a Mouse Model of Severe Aplastic Anemia, Leukemia. (2021) 35, no. 11, 3139–3151, 10.1038/s41375-021-01219-z. 33744909 PMC8849945

[22] Grazda R. , Seyfried A. N. , Maddipati K. R. , Fredman G. , and MacNamara K. C. , Resolvin E1 Improves Efferocytosis and Rescues Severe Aplastic Anemia in Mice, Cell Death & Disease. (2024) 15, no. 5, 10.1038/s41419-024-06705-7, 324. 38724533 PMC11082201

[23] Galipeau J. and Sensébé L. , Mesenchymal Stromal Cells: Clinical Challenges and Therapeutic Opportunities, Cell Stem Cell. (2018) 22, no. 6, 824–833, 10.1016/j.stem.2018.05.004. 29859173 PMC6434696

[24] Bär C. , Povedano J. M. , and Serrano R. , et al.Telomerase Gene Therapy Rescues Telomere Length, Bone Marrow Aplasia, and Survival in Mice With Aplastic Anemia, Blood. (2016) 127, no. 14, 1770–1779, 10.1182/blood-2015-08-667485. 26903545

[25] Li P. , Gong Z. , Shultz L. D. , and Ren G. , Mesenchymal Stem Cells: From Regeneration to Cancer, Pharmacology & Therapeutics. (2019) 200, 42–54, 10.1016/j.pharmthera.2019.04.005. 30998940 PMC6626571

[26] Zhao J. , Chen J. , and Huang F. , et al.Human Gingiva Tissue-Derived MSC Ameliorates Immune-Mediated Bone Marrow Failure of Aplastic Anemia via Suppression of Th1 and Th17 Cells and Enhancement of CD4^+^Foxp3^+^ Regulatory T Cells Differentiation, American Journal of Translational Research. (2019) 11, no. 12, 7627–7643. 31934306 PMC6943455

[27] Li Y. , Wang F. , and Guo R. , et al.Exosomal Sphingosine 1-Phosphate Secreted by Mesenchymal Stem Cells Regulated Treg/Th17 Balance in Aplastic Anemia, IUBMB Life. (2019) 71, no. 9, 1284–1292, 10.1002/iub.2035. 30889317

[28] Li Y. , Ren X. , and Zhang Z. , et al.Effect of Small Extracellular Vesicles Derived From IL-10-Overexpressing Mesenchymal Stem Cells on Experimental Autoimmune Uveitis, Stem Cell Research & Therapy. (2022) 13, no. 1, 10.1186/s13287-022-02780-9, 100. 35255957 PMC8900327

[29] Wang C. , Lv D. , and Zhang X. , et al.Interleukin-10-Overexpressing Mesenchymal Stromal Cells Induce a Series of Regulatory Effects in the Inflammatory System and Promote the Survival of Endotoxin-Induced Acute Lung Injury in Mice Model, DNA and Cell Biology. (2018) 37, no. 1, 53–61, 10.1089/dna.2017.3735. 29072959

[30] Meng X. , Li J. , and Yu M. , et al.Transplantation of Mesenchymal Stem Cells Overexpressing IL10 Attenuates Cardiac Impairments in Rats With Myocardial Infarction, Journal of Cellular Physiology. (2018) 233, no. 1, 587–595, 10.1002/jcp.25919. 28322445

[31] Kulasekararaj A. G. , Jiang J. , and Smith A. E. , et al.Somatic Mutations Identify a Subgroup of Aplastic Anemia Patients Who Progress to Myelodysplastic Syndrome, Blood. (2014) 124, no. 17, 2698–2704, 10.1182/blood-2014-05-574889. 25139356 PMC4383793

[32] Thol F. , Kade S. , and Schlarmann C. , et al.Frequency and Prognostic Impact of Mutations in SRSF2, U2AF1, and ZRSR2 in Patients With Myelodysplastic Syndromes, Blood. (2012) 119, no. 15, 3578–3584, 10.1182/blood-2011-12-399337. 22389253

[33] Yoshida K. , Sanada M. , and Shiraishi Y. , et al.Frequent Pathway Mutations of Splicing Machinery in Myelodysplasia, Nature. (2011) 478, no. 7367, 64–69, 10.1038/nature10496. 21909114

[34] Zhu C. , Lian Y. , and Wang C. , et al.Single-Cell Transcriptomics Dissects Hematopoietic Cell Destruction and T-Cell Engagement in Aplastic Anemia, Blood. (2021) 138, no. 1, 23–33, 10.1182/blood.2020008966. 33763704 PMC8349468

[35] Wei Y. , Zhang L. , and Chi Y. , et al.High-Efficient Generation of VCAM-1^+^ Mesenchymal Stem Cells With Multidimensional Superiorities in Signatures and Efficacy on Aplastic Anaemia Mice, Cell Proliferation. (2020) 53, no. 8, 10.1111/cpr.12862. PMC7445411 32597552

[36] Gholampour M. A. , Abroun S. , Nieuwland R. , Mowla S. J. , and Soudi S. , Mesenchymal Stem Cell-Derived Extracellular Vesicles Conditionally Ameliorate Bone Marrow Failure Symptoms in an Immune-Mediated Aplastic Anemia Mouse Model, Journal of Cellular Physiology. (2021) 236, no. 8, 6055–6067, 10.1002/jcp.30291. 33492726

[37] Li H. , Xu X. , and Wang D. , et al.miR-146b-5p Regulates Bone Marrow Mesenchymal Stem Cell Differentiation by SIAH2/PPARγ in Aplastic Anemia Children and Benzene-Induced Aplastic Anemia Mouse Model, Cell Cycle. (2020) 19, no. 19, 2460–2471, 10.1080/15384101.2020.1807081. 32840137 PMC7553565

[38] Akçay A. , Atay D. , and Erbey F. , et al.Safety and Efficacy of Co-Transplantation of Hematopoietic Stem Cells Combined With Human Umbilical Cord-Derived Mesenchymal Stem Cells in Children With Severe Aplastic Anemia: A Single-Center Experience, Experimental and Clinical Transplantation. (2022) 20, no. 12, 1114–1121, 10.6002/ect.2021.0027. 34142939

[39] Li R. , Tu J. , Zhao J. , Pan H. , Fang L. , and Shi J. , Mesenchymal Stromal Cells as Prophylaxis for Graft-Versus-Host Disease in Haplo-Identical Hematopoietic Stem Cell Transplantation Recipients With Severe Aplastic Anemia?—A Systematic Review and Meta-Analysis, Stem Cell Research & Therapy. (2021) 12, no. 1, 10.1186/s13287-021-02170-7, 106. 33541414 PMC7860635

[40] Guo X. , Tang Y. , and Zhang P. , et al.Effect of Ectopic High Expression of Transcription Factor OCT4 on the “Stemness” Characteristics of Human Bone Marrow-Derived Mesenchymal Stromal Cells, Stem Cell Research & Therapy. (2019) 10, no. 1, 10.1186/s13287-019-1263-4, 160. 31159871 PMC6547465

